# Editorial: Defect chemistry in electrocatalysis - volume II

**DOI:** 10.3389/fchem.2025.1613443

**Published:** 2025-04-25

**Authors:** Dafeng Yan, Longlu Wang, Feng Zeng, Huawei Huang

**Affiliations:** ^1^ College of Chemistry and Chemical Engineering, Hubei University, Wuhan, China; ^2^ College of Flexible Electronics (Future Technology), Nanjing University of Posts and Telecommunications (NJUPT), Nanjing, China; ^3^ State Key Laboratory of Materials-Oriented Chemical Engineering, College of Chemical Engineering, Nanjing Tech University, Nanjing, Jiangsu, China; ^4^ KAUST Catalysis Center (KCC), King Abdullah University of Science and Technology (KAUST), Thuwal, Saudi Arabia

**Keywords:** defect, electrocataiysis, energy storage and conversion, electrocatalysts, defect engineering

The second volume of the Defect Chemistry in Electrocatalysis series continues to explore the vital role of defect engineering in advancing electrocatalytic technologies (Yan et al., 2022a). Electrocatalysis is essential to the operation of devices used for electrochemical energy storage and conversion. Because the efficiency of these systems is heavily dependent on the kinetics of electrochemical reactions, developing high-performance electrocatalysts to accelerate these processes is crucial (Yan et al., 2023). Since these reactions primarily take place at the catalyst surface, the surface electronic structure significantly influences the catalytic activity (Xie et al., 2021). Defect engineering—through the intentional introduction of vacancies, dopants, or structural modifications—has emerged as a powerful strategy to tune catalyst behavior. By manipulating defects, researchers can enhance active site accessibility, modulate electronic structures, and improve reaction kinetics, ultimately boosting catalytic efficiency and selectivity (Yan et al., 2022b; Yan et al., 2019; Yan et al., 2017). In recent years, this area has witnessed rapid progress, with a surge of new research efforts emerging in response to the foundational studies presented in the previous Research Topic.

This Research Topic features recent advances that deepen our understanding of defect chemistry. Maseko et al. reveal that potassium and manganese co-promoters significantly enhance olefin selectivity in CoFe-ZSM-5 zeolites for CO_2_ hydrogenation, demonstrating the effectiveness of targeted metal modification in zeolite catalysts. Zhang et al. develop a nitrogen and boron dual-doped porous defect-rich carbon catalyst derived from saccharina japonica, achieving enhanced oxygen reduction activity. Their combined computational and experimental approach highlights the potential of biomass-based materials in sustainable electrocatalysis. Linling et al. review strategies for NO electroreduction to NH_3_, emphasizing the role of vacancy and doping defects in improving activity. Their work presents defect engineering as a viable strategy to achieve efficient electrocatalytic ammonia synthesis replacing the energy-intensive Haber-Bosch process. Chen et al. focus on the electrooxidation of 5-hydroxymethylfurfural, outlining how engineered anionic and cationic vacancies can accelerate reaction kinetics and improve catalyst performance for biomass valorization.

As guest editors, we extend our sincere gratitude to all the authors for their outstanding contributions, and to the reviewers for their insightful and constructive feedback. These contributions underscore the transformative impact of defect chemistry on electrocatalyst design. By elucidating the structure–performance relationship, this Research Topic advances the development of next-generation catalysts with greater efficiency, selectivity, and durability. Continued exploration in this field holds promise for breakthroughs in clean energy and environmental sustainability.
